# Theoretical Design of Dithienopicenocarbazole-Based Molecules by Molecular Engineering of Terminal Units Toward Promising Non-fullerene Acceptors

**DOI:** 10.3389/fchem.2020.580252

**Published:** 2020-11-05

**Authors:** Jie Feng, Hongshuai Wang, Nopporn Rujisamphan, Youyong Li

**Affiliations:** ^1^Jiangsu Key Laboratory for Carbon-Based Functional Materials & Devices, Institute of Functional Nano & Soft Materials (FUNSOM), Soochow University, Suzhou, China; ^2^King Mongkut's University of Technology Thonburi (KMUTT), Bangkok, Thailand

**Keywords:** organic solar cell, molecular engineering, density functional theory, dithienopicenocarbazole, non-fullerene acceptors (NFAs)

## Abstract

Dithienopicenocarbazole (DTPC), as the kernel module in A-D-A non-fullerene acceptors (NFA), has been reported for its ultra-narrow bandgap, high power conversion efficiency, and extremely low energy loss. To further improve the photovoltaic performance of DTPC-based acceptors, molecular engineering of end-capped groups could be an effective method according to previous research. In this article, a class of acceptors were designed via bringing terminal units with an enhanced electron-withdrawing ability to the DTPC central core. Their geometrical structures, frontier molecular orbitals, absorption spectrum, and intramolecular charge transfer and energy loss have been systematically investigated on the basis of density functional theory (DFT) and time-dependent density functional theory (TD-DFT) calculations. Surprisingly, NFA 4 highlights the dominance for its increased open circuit voltages while NFA 2, 7, and 8 exhibit great potential for their enhanced charge transfer and lower energy loss, corresponding to a higher short-circuit current density. Our results also manifest that proper modifications of the terminal acceptor with extensions of π-conjugation might bring improved outcomes for overall properties. Such a measure could become a feasible strategy for the synthesis of new acceptors, thereby facilitating the advancement of organic solar cells.

## Introduction

Currently, organic solar cells (OSCs), recognized for their great potential as sustainable energy sources, have been attracting extensive attention from researchers (Brabec et al., 2001; Gao et al., 2018; Upama et al., 2020; Zhao et al., 2020). OSCs have displayed unparalleled properties like practically inexhaustible resource storage, flexible modification, rapid power payback time, and being free of pollution during synthesis and operation (Lewis, 2007; Cheng et al., 2009; Lin et al., 2012). Fullerene and its derivatives have primarily been employed as acceptors and their widespread use has made significant contributions to the improved performance of OSCs (Sonar et al., 2011; Zhang et al., 2015). Nevertheless, despite much effort being devoted toward the development of fullerene derivatives, there still remain several troublesome obstacles to be overcome, such as complex synthesis, week absorption abilities, poor light harvesting properties, and hardly tunable band gaps (Liang and Yu, 2010; Lin et al., 2012; Chen et al., 2015). In recent years, the above difficulties have given birth to non-fullerene acceptor materials, which offer several unique advantages, including low-cost facile synthesis, tunable flexibility of FMO energy levels, and superior capacity of light absorption in the visible region (Anthony, 2011; Ala'a et al., 2014; Li et al., 2015a; Lin et al., 2015). However, researchers are still persisting in advancing progress in the research of non-fullerene acceptors (NFA).

Among numerous NFAs, the A-D-A type acceptors have received considerable attention from researchers (Li et al., 2019). After abundant exploitation and rapid development, A-D-A acceptors have proven to be a wise design to date, and are likely to become potential alternatives to fullerenes (Kini et al., 2020). In order to optimize the photovoltaic properties of OSCs, indacenodithienothiophene (ITs) have been widely used as central “D” cores to fabricate some small molecule NFAs. A typical molecule is ITIC, which can be used in discussions on photovoltaic performances of different polymer donor materials (Shen et al., 2018). And within various polymers, the cell of PBDTS-TDZ:ITIC offered the PCE of 13.35%, which is the best record in ITIC-based devices, as is reported by Peng's group (Xu et al., 2018). Moreover, Hou's group developed a new acceptor IT-4F via adding the fluorine atom to the IC “A” units and achieved an increased efficiency of 13.7% (Zhao et al., 2017). Another promising central core is BTP, as Yuan et al. demonstrated a remarkable PCE of over 15% for the novel acceptor BTP-4F, which is structured by end capping the BTP central “D” core with the 2F-DCI “A” units (Yuan et al., 2019). Later, Cui et al. replaced the fluorine atom at the terminal of BTP-4F with the chlorine atom to form a new acceptor, namely BTP-4Cl, and obtained a high efficiency of 16.5%, which is the highest among reported OSCs (Cui et al., 2019).

Recently, Yao et al. have synthesized a novel non-fullerene acceptor consisting of a highly electron-rich core, Dithienopicenocarbazole (DTPC), and two terminal electron-withdrawing units, 2-(5,6-difluoro-3-oxo-2,3-dihydro-1H-inden-1-ylidene) malononitrile (DFIC), which is proven to obtain considerably narrow gaps of 1.21 eV, quite high PCE up to 10.21%, and exceedingly low energy loss for only 0.45 eV simultaneously (Yao et al., 2018). However, DTPC-based acceptors would seem to be defective if compared to acceptors based on the core of IT and BTP, which are able to perform PCE over 13%, and still have more room for further research on higher PCE. Fortunately, as with A-D-A type acceptors, the central “D” core and the terminal “A” units are of equal importance and both of them play an important role in the overall performance of NFAs (Dey, 2019). In other words, besides the central core, end-capped groups may also affect the overall properties of photovoltaic devices to a great degree, and proper collocation of core and end groups could bring significantly improved outcomes (Shen et al., 2018). For instance, by molecular engineering of one more malononitrile group at the terminal, the efficiency highlights a noteworthy increase from 4.11% of FBR to 8.4% of BAF-4CN (Holliday et al., 2015; Gupta et al., 2017). Additionally, previous research has suggested that the device performance of different NFAs would vary depending upon the types of end-capped units according to their electron-withdrawing capability (Singh and Prakash, 2019), and extending the π-conjugation and enhancing the electron-withdrawing ability of terminal groups may be effective and feasible to promote the photovoltaic properties (Song et al., 2020). So here we pay our attention to suitable molecular engineering of extended π-conjugation and enhanced electron-withdrawing ability at the terminal units to modulate photophysical performance of NFAs.

**Figure 1:**
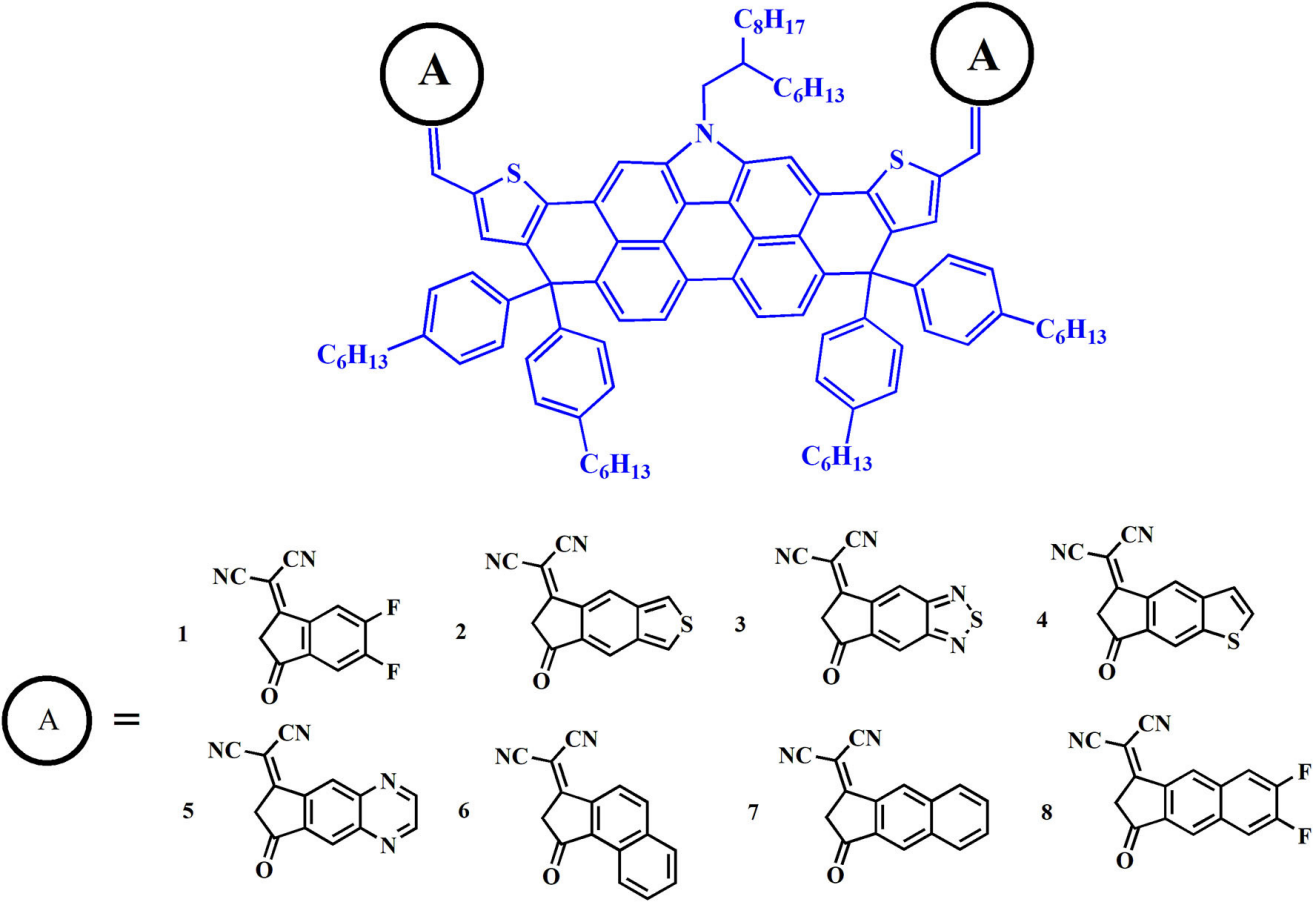
Molecular structures of the investigated acceptors.

Herein, we express a keen interest in DTPC-based molecules and focus on expanding the electron-withdrawing ability of terminal units to investigate the photovoltaic properties. It could be reasonably assumed that such a modification may not only maintain the original forte of narrow band gaps and low energy loss benefitting from the electron-rich core, but also possess a higher open circuit voltage and short circuit current, corresponding to improved PCE. In this article, we take DTPC-DFIC for reference (NFA 1) to innovatively design a series of new non-fullerene acceptors by the addition of enhanced electron-withdrawing terminal units to a DTPC core, as is shown in Figure 1. By means of density functional theory (DFT) and time-dependent DFT (TD-DFT) computational methodology, geometrical structures, frontier molecular orbitals, and absorption spectrum are systematically investigated, then open circuit voltage, energetic driving force, and energy loss are reliably predicted. The simulation of intramolecular charge transfer, charge transport, and stability properties are also covered in this article. Exploration of the above physical parameters closely correlated with PCE permits us to fully understand how such measures influence the photovoltaic performance of the device, and to seek potential alternatives to previous DTPC-based acceptors.

## Theoretical Methods and Computational Details

There is no doubt that side alkyl chains have a notable impact on molecular aggregation and geometrical structures, hence they were considered for the investigated NFAs during the calculations. And for the sake of obtaining as accurate results as possible, all tasks in this article were carried out with the 6-31G(d,p) basis set. In order to increase the reliability of calculated value, three DTF functionals with different HF_exc_ were used to calculate the highest occupied molecular orbital (HOMO) level of NFA 1, including B3LYP, PBE0, and MPW1K (Becke, 1993; Stephens et al., 1994; Adamo and Barone, 1999; Lynch et al., 2000). As can be seen in Supplementary Table 1, the result (−5.36 eV) calculated by PBE0 function showed great accordance with experimental data (−5.31 eV), so DFT/PBE0 was selected to perform ground-state-related calculations (Yao et al., 2018). The cationic and anionic geometries were also optimized at the PBE0 level to further evaluate IPs, EAs, λ_e_, and λ_h_. The absorption spectrum of investigated NFAs were simulated by TD-DFT/CAM-B3LYP under the solvent condition of dichloromethane with polarizable continuum model (PCM), as CAM-B3LYP (19% HF_exc_ at short-range and 65% HF_exc_ at long-range) was proven to be a valid function that could exactly take the effect of charge transfer into account (Gross and Kohn, 1985; Yanai et al., 2004). The charge density difference (CDD), electron–hole distributions, and inter fragment charge transfer (IFCT) were implemented in Multiwfn 3.7 package to understand intramolecular electronic transition (Lu and Chen, 2012). Incidentally, the donor molecule PBT7-Th was simplified into repeated units (PBT7-Th)_3_ to save computational costs, as is shown in Supplementary Figure 1, and its geometrical structure was optimized at the B3LYP/6-31G(d,p) level and demonstrated in Supplementary Figure 2. All the calculations above were performed using the Gaussian 09 software package (Frisch et al., 2013).

## Results and Discussion

### Geometrical Structure

**Figure 2:**
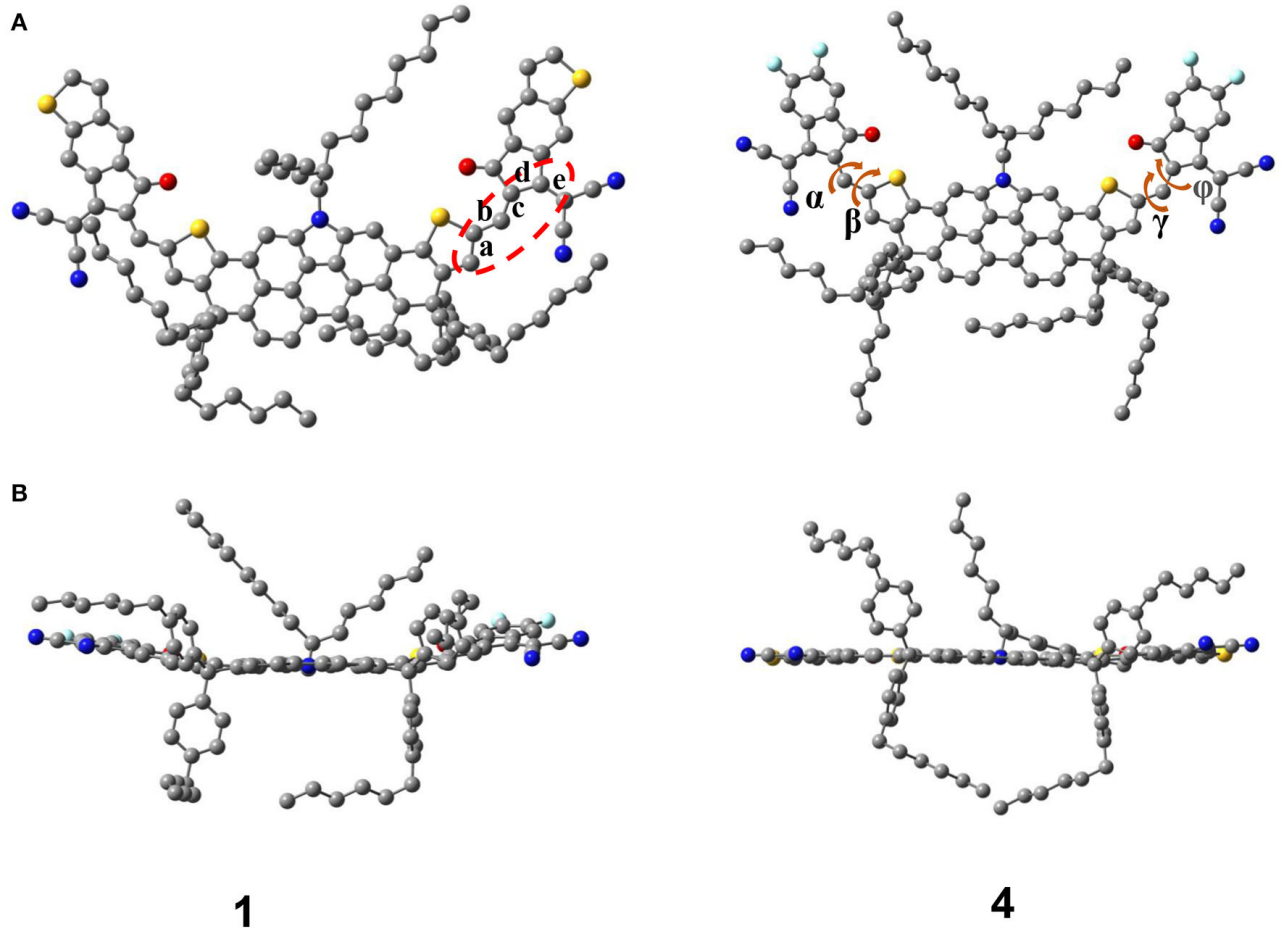
Optimized structure of NFA 1 and 4 from the top view **(A)** and side view **(B)**.

Without any structural constraints, all ground-state geometrical structures of NFAs are optimized and shown in Supplementary Figure 3. Taking NFA 1 and 4 as example, we show their optimized structure from the top view and side view in Figure 2. The dihedral angles and bond lengths between the DTPC core and end-capped acceptor are listed in Supplementary Table 2. Obviously, all A-π-A type NFAs based on the DTPC core are presented as quasi-planar conformations. All dihedral angles of investigated NFAs are extremely small, mostly close to zero. Taking molecular vibration when optimizing into consideration, the distortion could be overlooked, and excellent planarity is achieved between the core and acceptor. As is known, the core of DTPC plays a significant role in expanding conjugate planes of molecules when used for acceptors. Fortunately, for all A-D-A skeletal structures investigated, large planarity not only benefits from the DTPC core, but also relies on the coplanarity of the core and acceptor. Improvement of the planarity of NFAs can effectively narrow the energy gap and broaden the optical absorption range of NFAs, which also enhances the electron mobility of NFAs. After extending π-conjugation of terminal groups, the molecular structures still maintain superior conjugation and coplanarity, which could lead to narrow band gaps and excellent optical properties.

Bond length alternation (BLA) is a construct that could be applied to describe the conjugate degree of a molecule, which is defined as the average bond length difference between a single bond and double bond in a chain, as is calculated by the equation below (Meyers et al., 1994; Lee et al., 2001; Yang and Kertesz, 2006; Wang et al., 2014):

(1) $$\begin{array}{l} \text{BLA }=\frac{\text{b}+\text{d}}{2}\text{-}\frac{a+c+e}{3} \end{array}$$

Table 1 shows the bond length of a, b, c, d, e, and BLA values for neutral, anion, and cation. Values of bond length range from 1.374 to 1.461 Å, which is in the middle of C–C bonds (1.54 Å) and C=C bonds (1.33 Å). Therefore, several bonds measured are not typical single or double bonds. BLA values of neutral are obviously larger than anion, but a little smaller than cation, indicating that all molecules are suitable for acceptors rather than donors. Due to good conjugation of electronic structure, NFAs prefer to be changed into an anionic state when delocalized. Further, larger BLA indicates larger differences between bonds and higher energy levels. The neutral BLA values of NFA 4 (0.0537 Å), 6 (0.0549 Å), and 7 (0.0523 Å) are a little higher than those of others, which could be conducive to raising their LUMO level. And the differences between neutral (0.0537 Å/0.0549 Å) and anionic state (0.0204 Å/0.0211 Å) of NFA 4 and 6 are slightly larger, which could make exciton delocalization better and further to make electron transport more favorable. Therefore, molecular engineering of terminal units will change the conjugate degree of the whole structure slightly, and some of these changes would have very positive results.

**Table 1:** Selected bond lengths (Å) and BLAs (Å) of the investigated NFAs.

**NFAs**	**a**	**b**	**c**	**d**	**e**	**BLA (neutral)**	**BLA (anion)**	**BLA (cation)**
1	1.399	1.414	1.377	1.460	1.379	0.0519	0.0200	0.0696
2	1.397	1.414	1.378	1.461	1.383	0.0515	0.0248	0.0681
3	1.399	1.412	1.380	1.459	1.381	0.0483	0.0276	0.0666
4	1.397	1.415	1.376	1.460	1.382	0.0537	0.0204	0.0700
5	1.400	1.413	1.379	1.459	1.380	0.0495	0.0230	0.0676
6	1.395	1.417	1.374	1.458	1.379	0.0549	0.0211	0.0707
7	1.397	1.415	1.377	1.461	1.382	0.0523	0.0226	0.0692
8	1.398	1.414	1.377	1.460	1.382	0.0512	0.0221	0.0689

### Frontier Molecular Orbital

**Figure 3:**
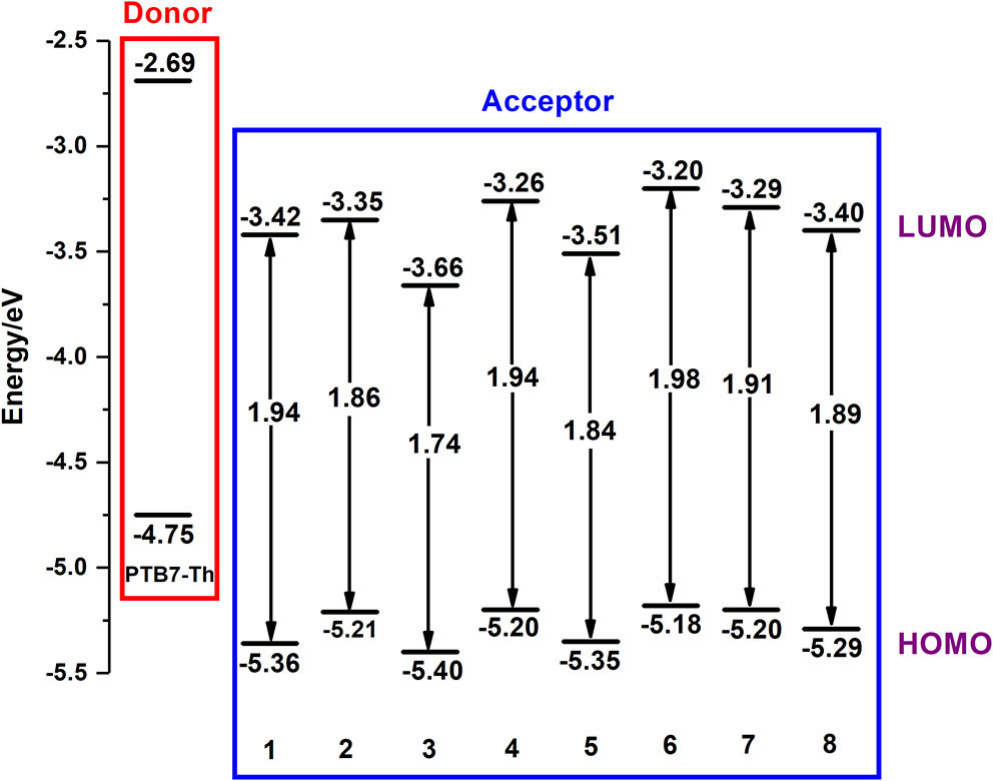
Orbital energy level diagrams for investigated NFAs.

It is essential to study the highest occupied molecular orbital (HOMO) and lowest unoccupied molecular orbital (LUMO) of any organic molecule, because frontier molecular orbital (FMO) exerts conclusive effects on open circuit voltages and plays an extremely important role in the property of excited-state and optical performance (Ku et al., 2011; Li, 2012; Janssen and Nelson, 2013). All FMO energy levels of investigated NFAs are depicted in Figure 3.

Clearly, all NFAs perform perfect adaptation to the donor PTB7-Th, ensuring minimal energy loss on electron transfer and as high an internal voltage as possible, and the introduction of an electron-withdrawing group changes their properties of energy levels greatly (Nielsen et al., 2015). Compared with NFA 1, NFA 2, 4, and 6–8 possess higher LUMO and HOMO energy levels, while NFA 3 and 5 possess narrower band gaps. Apparently, LUMO of NFA 3 is lower than that of 2 and LUMO of NFA 5 is lower than that of 7. By contrast, their band gaps show the opposite tendency. Therefore, we infer that the introduction of nitrogen, which is usually considered as an atom with strong electronegativity, may lower LUMO values, although it could lead to a decrease in the energy gap. With comparisons of NFA 7 and 8, it is reasonable to deduce that introducing fluorine and chlorine could slightly narrow the band gap, which is consistent with experimental results. Hence, for A-π-A skeletal NFAs, proper modifications of the terminal acceptor could be accompanied with relatively high LUMO, further effectively increasing the value of open circuit voltages. However, excessive π-conjugations at end-capped acceptors may not always be a good choice. Instead, it could conspicuously decrease the LUMO further to have a negative impact on open circuit voltages.

**Figure 4:**
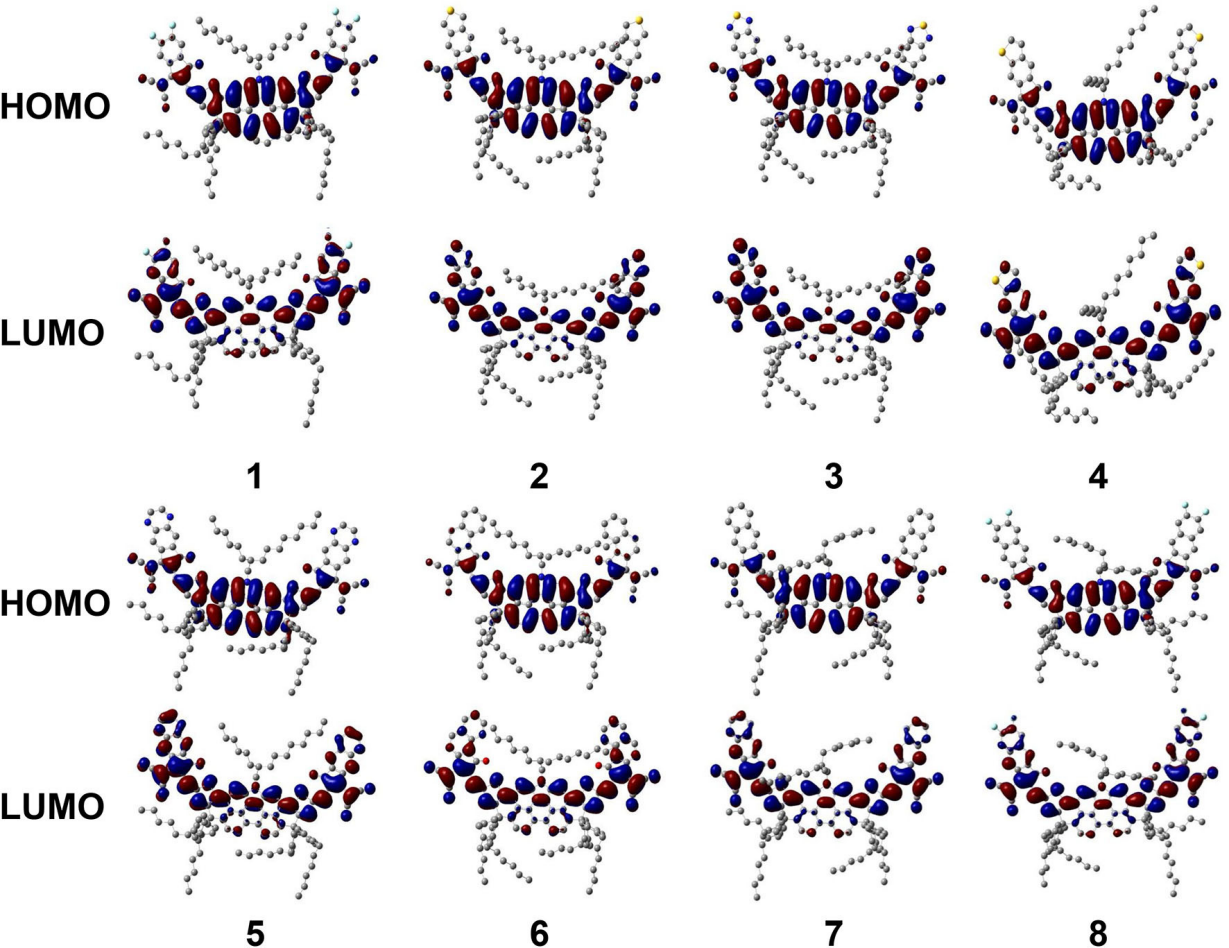
Frontier molecular orbital diagram for investigated NFAs.

To more wholly understand the factors that cause the difference in FMO levels, all NFAs are divided into three fragments, a core of DTPC and two end-capped groups, to calculate respective contributions to HOMO and LUMO. The results are collected in Supplementary Tables 3, 4, and FMOs of all NFAs are illustrated in Figure 4.

Combining graphic analysis and numerical comparison, we conclude that for HOMO, DTPC-core takes hold observably because its percentage of contributions is more than 80% while other moieties only sum up to occupy ~15%. And outcomes present significant variations in how much the three fragments contribute to LUMO medially, as the percentages are all around 30–40%. So, we can reasonably consider that HOMO of NFAs mainly come from the DTPC core while LUMO of NFAs are distributed in the whole molecule. Additionally, from these FMO figures, we are able to observe that for all NFAs, both the HOMO orbital and the LUMO orbital present π and π^*^ features, which will promote charge transfer. It is also verified that resulting from its large conjugate planar peculiarity, DTPC is a prominent selection to be utilized as the core of narrow-band gap NFAs due to its favorable abilities to withdraw electron and holes. Last but not least, with the exploration of FMO distribution, we testify that it is feasible to raise LUMO energy levels by adjusting suitable end-cap acceptors.

### Open Circuit Voltages and Energetic Driving Force

As we know, maximizing open circuit voltages (V_OC_) properly is commonly applied to facilitate the efficiency of OSCs, thus V_OC_ is an indispensable parameter to examine the performance of photovoltaic devices, which is usually calculated using the equation (Scharber et al., 2006; Rand et al., 2007; Credgington and Durrant, 2012):

(2) $$\begin{array}{l} V_{OC}\;=\;\frac{1}{\text{e}}\left(\left|E_{HOMO}^{D}|-|E_{LUMO}^{A}\right|\right)-\Delta V \end{array}$$

where $E_{HOMO}^{D}$ represents the HOMO energy level of the donor, while $E_{LUMO}^{A}$ represents the LUMO energy level of acceptor, and Δ*V* is considered as exciton binding energy for charge separation. As for non-fullerene acceptors, it is empirically referred to as 0.5 eV (Pan et al., 2016; Yin et al., 2018).

Energetic driving force (Δ*E*_*L*−*L*_) is another parameter to estimate whether a molecule is ideal for application in the device or not. Δ*E*_*L*−*L*_ is equal to the LUMO difference between donor and acceptor, which is intuitionally expressed by the formula (Faist et al., 2012):

(3) $$\begin{array}{l} \Delta E_{L-L}=|E_{LUMO}^{A}|-|E_{LUMO}^{D}| \end{array}$$

With the assistance of FMO energy levels discussed above, we were able to obtain the two dominant factors of all NFAs, which are demonstrated in Table 2. Firstly, the calculated V_OC_ of NFA 1 (0.83 eV) is quite close to the experimental measured value (0.77 eV), which attests that our consequences are reliable in accordance with actual research (Yao et al., 2018). Furthermore, most V_OC_ values of NFAs studied are higher than that of NFA 1, except for NFA 3 and 5. These NFAs with prior V_OC_ may have great potential for being good candidates. From Table 2, the values of Δ*E*_*L*−*L*_ are all higher than 0.3 eV, which ensures an adequate driving force for separated charges to transfer between donor and acceptor (Zhan and Yao, 2016). However, it is not negligible that a superfluous driving force does not mean a better performance. Several previous pieces of research have substantiated that there will be excess energetic loss degenerating the efficiency of the device if the value is more than 0.8 eV (Li, 2012), so NFA 3 and 5 could not be regarded as advisable acceptors in view of their unsuitable driving force. To conclude, unfortunately on account of an inferior performance on open circuit voltages and energetic driving force, NFA 3 and 5 will be seldom mentioned below in this article. In addition, more π-conjugations at end-capped units does not mean high open circuit voltages, so in order to improve V_OC_, the balance should be found. And according to the results, the structural modification at terminal groups like NFA 2, 4, 6, and 7 may be admissible.

**Table 2:** Calculated open circuit voltages *V*_*OC*_ (eV), energetic driving force Δ*E*_*L*−*L*_ (eV) for investigated NFAs.

**NFAs**	**1**	**2**	**3**	**4**	**5**	**6**	**7**	**8**
*V*_*OC*_	0.83	0.9	0.59	0.99	0.74	1.05	0.96	0.85
Δ*E*_*L*−*L*_	0.73	0.66	0.97	0.57	0.82	0.51	0.60	0.71

### Absorption Spectra

**Figure 5:**
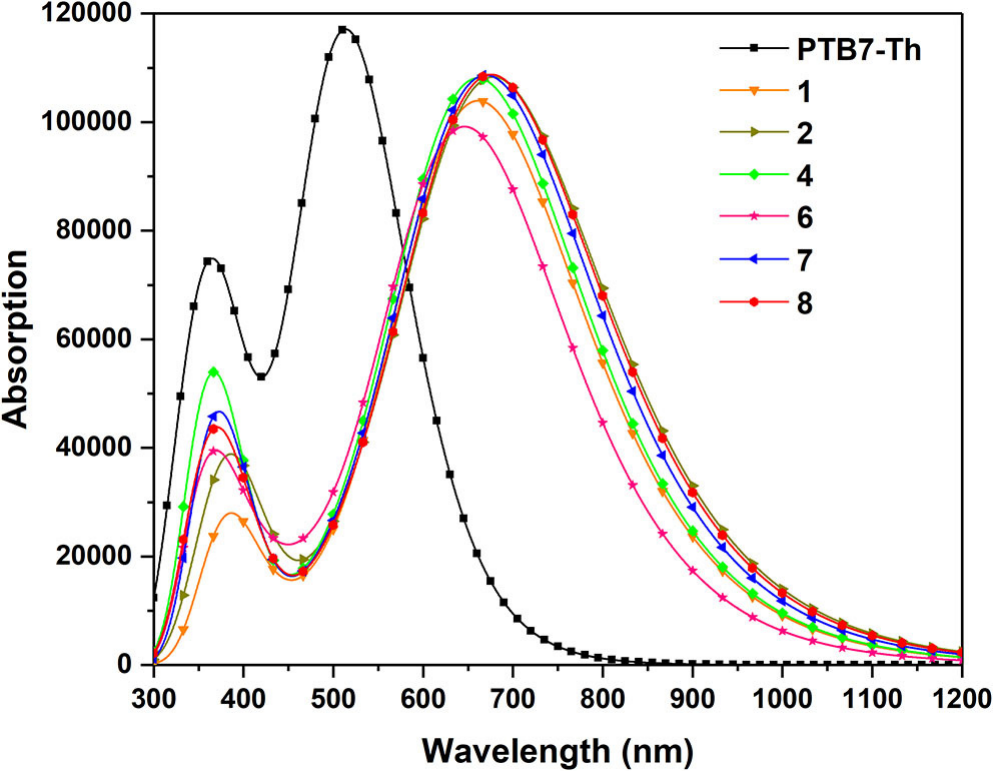
Simulated absorption spectra for investigated NFAs.

As far as we know, in addition to open circuit voltage, another crucial parameter affecting the efficiency of OSCs is short-circuit current density (J_SC_), which is determined by photo-physical processes and photoexcitation properties to a considerable extent (Mühlbacher et al., 2006; Murphy and Frechet, 2007). In this case, it is a requisite for non-fullerene acceptors to possess wide absorption spectra matching well with that of donor molecules and solar spectrums (Nielsen et al., 2015; Sui et al., 2017). Herein, we further explored the absorbing ability of NFAs in order to seek the influence of substituting different π-conjugate units at the terminal group compared with parent molecules. Accordingly, we figure out the simulated absorption spectra in Figure 5 and summarized the excitation energy, maximum absorption peaks, largest oscillator strength, light harvesting efficiency, normalized integrated spectral area, and major configurations in Table 3. The normalized integrated spectral area is defined as the proportion of the integrated spectral area of other NFAs to that of NFA 1 in the whole absorption range, and light harvesting efficiency is calculated as ηλ = 1-10^-f^, where f corresponds to the highest oscillator strength (Song et al., 2020).

**Table 3:** The excitation energies (eV), associated wavelengths (nm), oscillator strengths, light harvesting efficiency, normalized integrated spectral area, and orbital contribution.

	**State**	**E**	**λ_max_**	**f**	**ηλ**	**S**	**Major configurations**
1	S_1_	1.86	666	2.50	0.997	1	HOMO→ LUMO (83.5%)
2	S_1_	1.81	686	2.57	0.997	1.15	HOMO→ LUMO (83.5%)
4	S_1_	1.86	666	2.59	0.997	1.11	HOMO→ LUMO (83.2%)
6	S_1_	1.90	652	2.37	0.997	0.98	HOMO→ LUMO (80.6%)
7	S_1_	1.83	677	2.59	0.997	1.15	HOMO→ LUMO (83.7%)
8	S_1_	1.82	682	2.59	0.997	1.15	HOMO→ LUMO (83.7%)

From Figure 4, the broad absorption of NFAs ranges from 300 to 900 nm and highly overlaps with the visible spectrum and near infra-red spectrum. Delightfully, all NFAs exhibit perfect matching for the donor due to wider absorption in the area of 600–800 nm, where PTB7-Th presents a weak absorption. Meanwhile, PTB7-Th makes up for the shortfall that NFAs have in deficient absorption in the region of 450–600 nm. Compared with the absorption wavelength of NFA 1, NFA 4, 7, and 8 show slight bathochromic shifts, while NFA 6 shows hypsochromic shifts of 14 nm. More importantly, the values of the normalized integrated spectral area show similar tendencies that most NFAs, other than NFA 6, have larger spectral areas which means an enhanced capacity for light absorbing. The oscillator strengths at the maximum absorption wavelengths are sorted in the order of 4 = 7 = 8 = 2 > 1 > 6, and, generally, higher oscillator strength is related to better absorption coefficients and transition probabilities. Hence, NFA 2, 4, 7, and 8 are proven to be hopeful for high-efficient acceptors in terms of their admirable absorbing ability The excitation energies are in the order of 6 > 1 = 4 > 7 > 8 > 2, excellently corresponding to the sequence of band gap value. In consequence, we deduce that the broader band gap of NFA 6 degenerates its optical performance, including absorption peak, spectral area, and oscillator strength. Inspection of Table 3 reveals that the strongest absorption peak is originated by S0→ S1 electronic transitions derived from HOMO to LUMO. Learning from the FMO discussion above, we conclude that HOMO mainly distributes in the core of the molecule while LUMO spreads in the whole molecule, and both exhibit π and π^*^ orbital features. As a result, the transition nature of main absorption could be assigned to π-π^**^ transition with local excitation and charge-transfer excitation.

Consequently, NFA 2, 4, 7, and 8 with intense absorption spectra and superior sunlight-absorbing ability tend to have enhanced short-circuit current density and be advantageous candidates, which could be due to adopting different terminal π-conjugation groups properly. It implies that suitable molecular engineering of end-capped units significantly affects the optical absorption of the designed acceptors and could be an effective strategy to enhance the short-circuit current density and further enlarge the photovoltaic energy conversion of the device. Unremarkable performance on molecular absorption and charge transfer ability of NFA 6 may be attributed to its special configuration, indicating that such a molecular π-conjugation at the end-cap group is inadaptable for this system, and NFA 6 would not be discussed in detail in this article.

### Energy Loss

**Figure 6:**
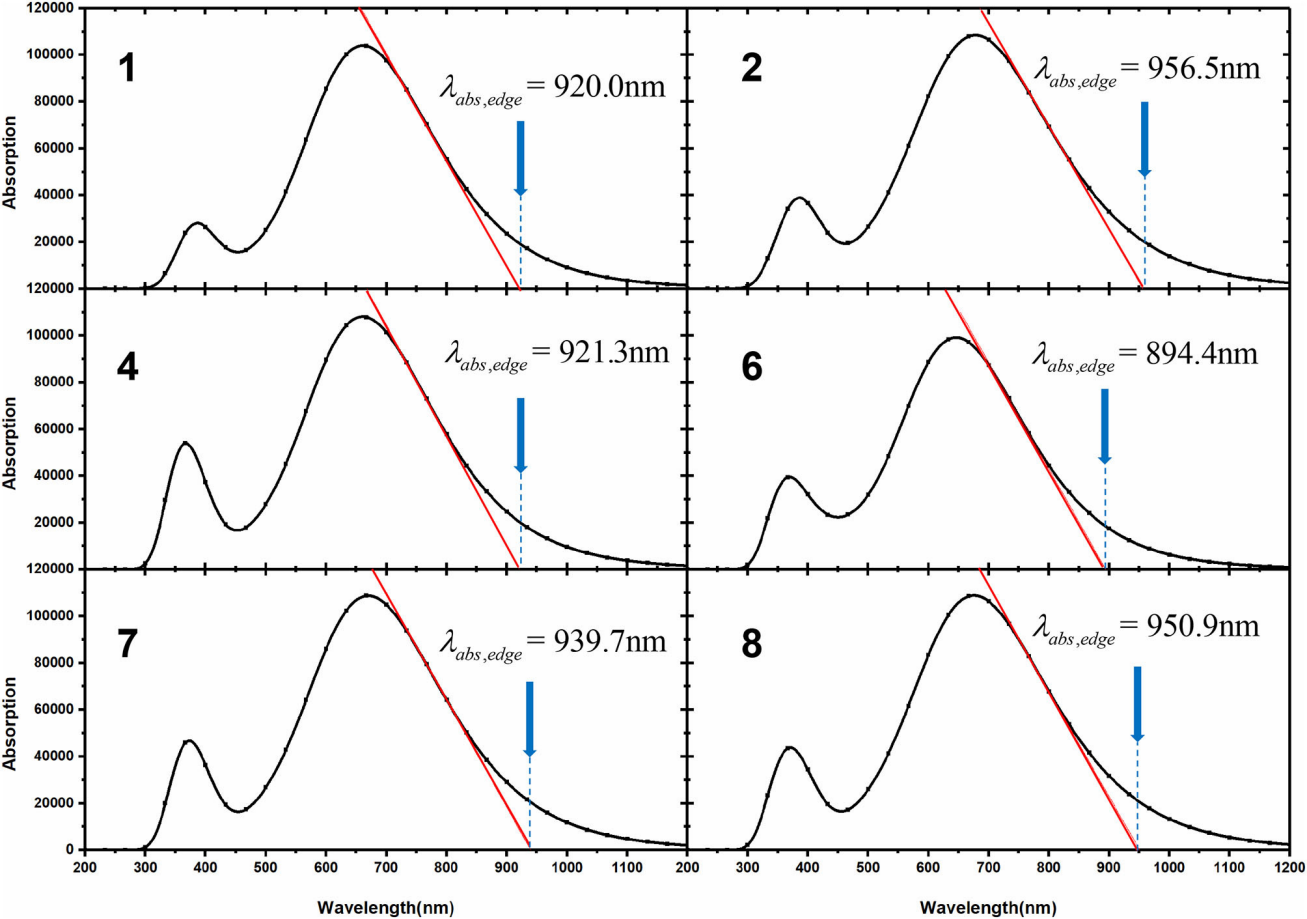
Calculated λ_*abs,edge*_ for investigated NFAs.

According to previous research, NFA 1 is considered as a non-fullerene acceptor with high power conversion efficiency and low energy loss. Evidently, energy loss (*E*_*loss*_) is a vital factor to estimate the capacity of the device and it is encouraging to obtain an *E*_*loss*_ value as small as possible. For the sake of thorough analysis on the cell performance of designed acceptors, we calculate energy loss of investigated NFAs corresponding to the equation below (Veldman et al., 2009; Li et al., 2015b):

(4) $$\begin{array}{l} E_{loss}=E_{g}^{opt}-qV_{OC} \end{array}$$

where V_OC_ represents open circuit voltage (which has been discussed above) and $E_{\text{g}}^{opt}$ represents a relatively smaller optical band gap of donor and acceptor. According to experimental results, NFA based on DTPC-core exhibits an extremely small band gap, thus we employed the optical gap of NFA and calculated it by the following formula:

(5) $$\begin{array}{l} E_{\text{g}}^{opt}\approx \frac{1240}{\lambda_{abs,edge}} \end{array}$$

where λ_*abs,edge*_ is equal to absorption edge wavelength, which is depicted in Figure 6, and parameters with respect to energy loss are collected in Table 4.

**Table 4:** Calculated λ_*abs,edge*_ (nm), $E_{g}^{opt}$ (eV), and *E*_*loss*_ (eV) for investigated NFAs.

**NFAs**	**1**	**2**	**4**	**7**	**8**
λ_*abs,edge*_	920.9	956.5	921.3	939.7	950.9
$E_{g}^{opt}$	1.35	1.30	1.35	1.32	1.30
*E*_*loss*_	0.52	0.4	0.36	0.36	0.45

Compared with NFA 1, four designed molecules all possess smaller *E*_*loss*_ values owing to their lower $E_{g}^{opt}$ and higher V_OC_. Indeed, *E*_*loss*_ of NFA 1 may be overrated as the calculated value (0.52 eV) is slightly higher than the experimental data (0.45 eV), indicating that other NFAs would demonstrate better performance in reality than in theoretical prediction (Yao et al., 2018). As a rule, any extra loss of energy is generally connected to thriftless driving force and inefficient charge transfer that affects the PCE of the device negatively. Hence, it may be promising to utilize the designed acceptors to improve the performance of OSCs due to the disappearance of limitation by large energy loss, and extended electron-withdrawing groups at the terminal may become favorable to decrease energy loss for non-fullerene molecules.

### Intramolecular Charge Transfer

**Figure 7:**
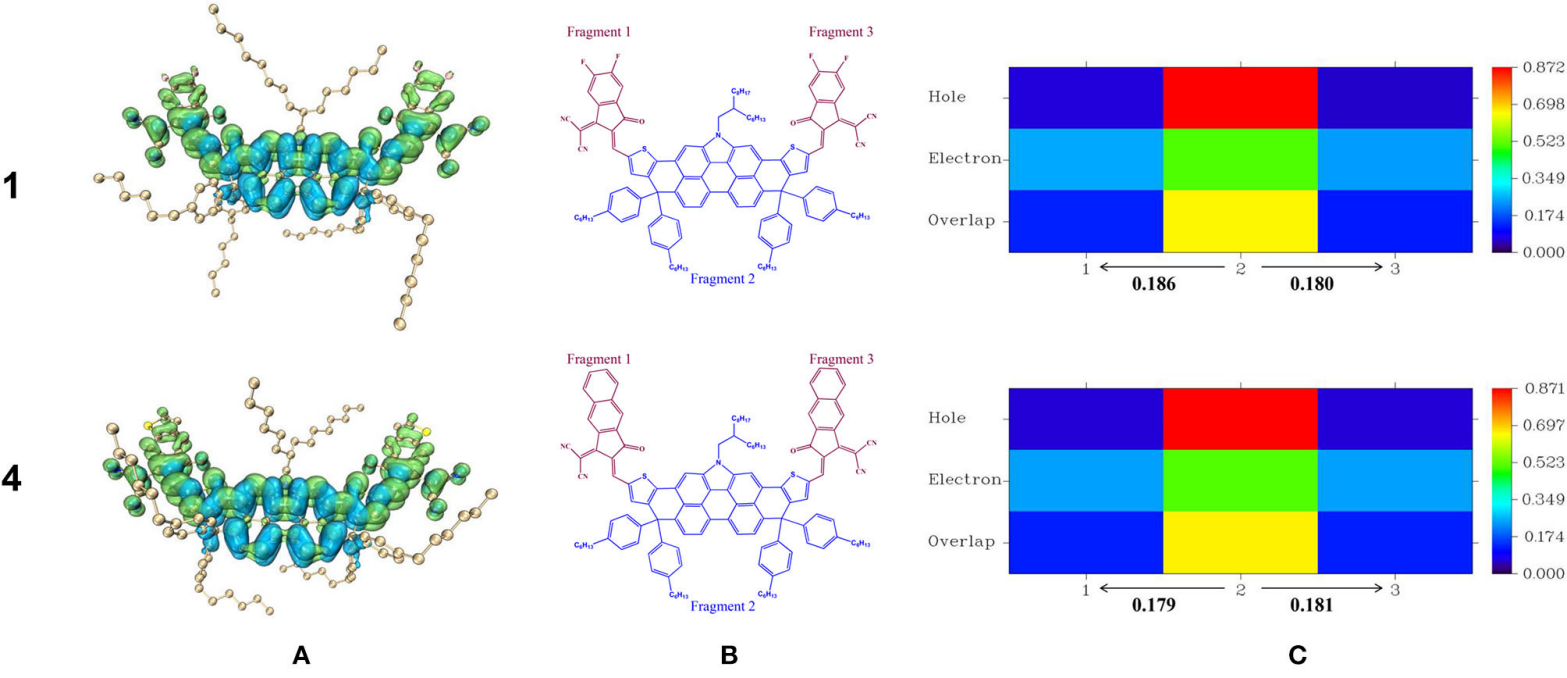
Hole and electron distribution for the S0→ S1 transitions of NFA 1,4 **(A)**, and heat maps associated with S0→ S1 transitions for hole–electron overlap in various fragment of NFA 1,4 **(B,C)**.

Photoexcitation always accompanies exciton dissociation to burst the restraints of Coulomb attraction (Lemaur et al., 2005; Clarke and Durrant, 2010). With the intention of characterizing exciton properties during the excitation process, we paid attention to the study of intramolecular charge transfer (Sui et al., 2019). Here we selected NFA 1 and 4 as examples and drew their hole and electron distribution for the S0→ S1 transitions in Figure 7A, then we partitioned it into three fragments and applied the inter fragment charge transfer (IFCT) method to calculate the actual amount of charge transfer, as is shown in Figures 7B,C. The results of other NFAs are available in Supplementary Figure 4, and concrete data associated with the excitation process is collected in Supplementary Table 5. From Figure 7A, it is visualized to discover that holes mainly concentrate on the center of the molecule while electrons localize along the whole molecule. To be exact, the terminal units are almost fully occupied by electrons. That is to say, during the process of photoexcitation, partial electrons are photoinduced and delocalized from the center of the molecule to end-capped group, leading to the generation of holes at the DTPC-core, revealing typical charge-transfer (CT) excitation feature. Meanwhile, some electrons are photoinduced locally at the center of the molecule, presenting localized excitation (LE) feature. Consequently, it is confirmed that photoexcitation of the designed molecule is displayed in the form of both charge-transfer excitation and localized excitation, or rather π-π^**^ transition, which is in exact accordance with the analysis above. It can be accurate to verify the above-mentioned conclusion and further quantify the charge transfer from Figure 7C that fragments 2 (DTPC) provide nearly equivalent electrons for fragments 1 and fragments 3, ~0.18 electrons. This can be attributed to the A-D-A skeletal structures in which DTPC holds a strong electron-rich property, profiting from its large conjugate planes, while end-capped units are electron-withdrawing. As a result, we believe that electron-withdrawing substituents at the terminal group may significantly affect its interaction with DTPC-core in the excited states and change the possibility of the effective exciton dissociation.

**Figure 8:**
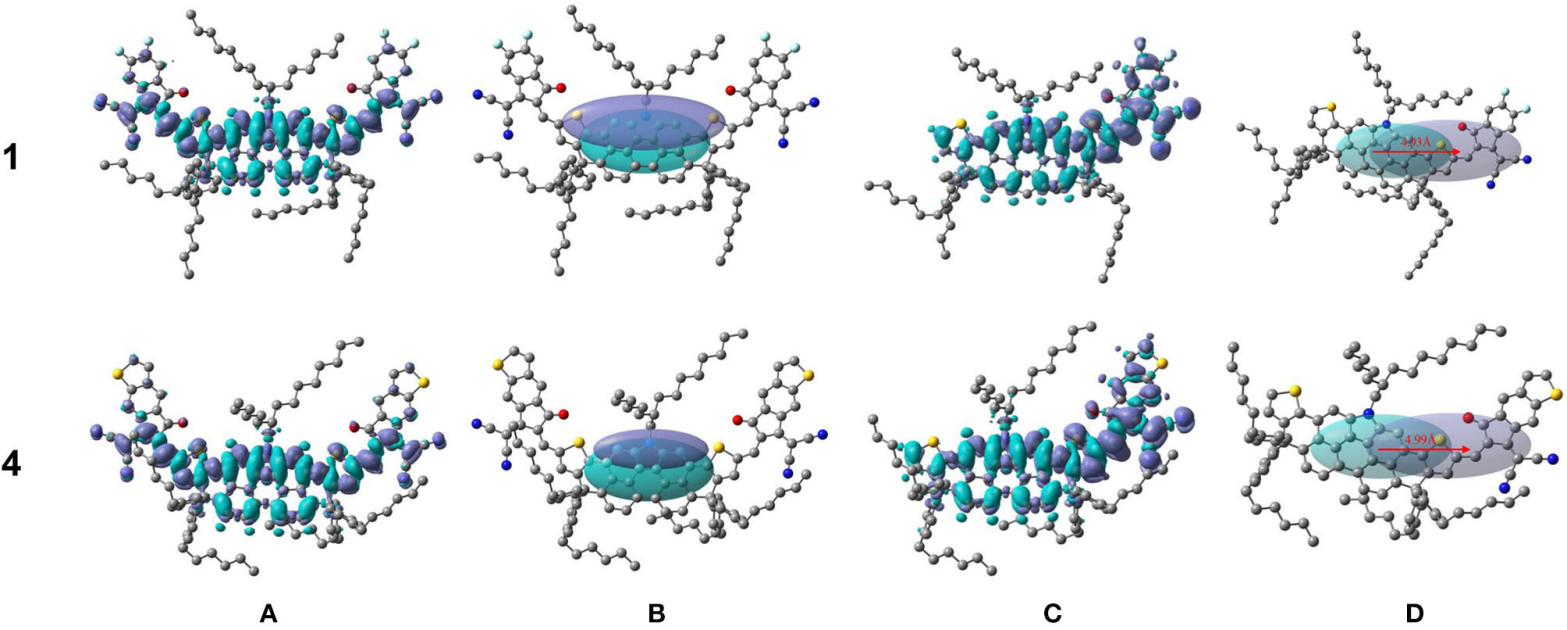
Charge density differences Δρ between ground state and first excited state and charge transfer distance D_CT_ (red arrows) for NFA 1 and 4 **(A,B)** and half of NFA 1 and 4 **(C,D)**.

Additionally, it is in our interest to deeply explore how the end-capped group affects the intramolecular charge transfer by charge density difference (CDD) maps. Firstly, we figured out CDD maps of NFA 1 and 4 between ground state and first excited state in Figure 8A, where the violet and turquoise colors stand for an increase and a decrease in electron density, respectively. Then we focused attention on the charge-transfer length (D_CT_) which represents the central distance between two regions of density decrease and increase, as is exhibited in Figure 8B. It is worth noting that, for the NFAs investigated, the barycenters of the positive electron density and negative electron density are both positioned at the core of DTPC owing to their central symmetric structure, making it difficult to describe the charge-transfer length exactly. Fortunately, there have been several previous researches selecting D_CT_ of the half-molecule on behalf of the charge-transfer length, offering a novel approach to address the problem (Wang et al., 2015; Chen et al., 2017; Song et al., 2020). Utilizing their experience for reference, we chose half-molecules of NFA 1 and 4 as the subject and figured out their charge density differences map and charge-transfer length in Figures 8C,D. The CDD maps and D_CT_ in half-molecules of other NFAs can be achieved in Supplementary Figure 5, and relevant values, including q, D_CT_, and variation in dipole moment Δμ_half_, are listed in Table 5.

**Table 5:** Transferred charge q(|*e*^−^|) for NFAs and *q*_half_(|*e*^−^|), charge-transfer distance *D*_half_(Å), variation in dipole moments Δ*μ*_half_ (D) for the transition S0→ S1 for half-NFAs.

**NFAs**	**1**	**2**	**4**	**7**	**8**
q	0.73	0.77	0.75	0.76	0.76
*q*_half_	0.80	0.84	0.82	0.83	0.83
*D*_half_	4.93	5.14	4.99	5.09	5.09
Δ*μ*_half_	19.03	20.82	19.50	20.30	20.38

There is no doubt that the designed acceptors all possess slightly larger values of q, D_CT_, and Δμ_half_ compared to NFA 1 from Table 5, proving the deduction that the capacity of charge transfer increases with the addition of electron-withdrawing ability at the terminal group. Particularly, profiting from enhanced intramolecular charge transfer, NFA 2, 7, and 8 may prominently overshadow other NFAs in the aspect of photovoltaic performance. To sum up, with additional photocurrent contributions, NFA 2, 7, and 8 may be good at the charge-transfer properties upon photoexcitation, thereby increasing the short circuit current, and introduction of π-conjugated units at the terminal group could become a considerable way to improve the J_SC_ of a device.

### Charge Transport and Stability Properties

For the purpose of estimating charge transport and stability properties, we summarize electron affinities (EAs), ionization potentials (IPs), reorganization energies of electron (λ_e_) and hole (λ_h_), and absolute hardness (η) in Table 6.

**Table 6:** Calculated molecular IP, EA, λ_*e*_, λ_*h*_, and *η* (all in eV) for investigated NFAs.

**NFAs**	**IP**	**EA**	λ_*e*_	λ_*h*_	*η*
1	5.93	2.85	0.18	0.17	1.54
2	5.77	2.79	0.13	0.15	1.49
4	5.77	2.74	0.18	0.15	1.52
7	5.76	2.74	0.15	0.15	1.51
8	5.83	2.89	0.18	0.15	1.47

According to Marcus theory, the rates of charge transfer are greatly determined by λ_e_ and λ_h_, which could be calculated by the equation below (Marcus, 1993; Köse et al., 2007):

(6) $$\begin{array}{l} \lambda_{\text{e}}=\left(E_{0}^{-}-E_{-}^{-}\right)+\left(E_{-}^{0}-E_{0}^{0}\right) \end{array}$$

(7) $$\begin{array}{l} \lambda_{h}=\left(E_{0}^{+}-E_{\text{+}}^{+}\right)+\left(E_{+}^{0}-E_{0}^{0}\right) \end{array}$$

where $E_{0}^{-}$($E_{0}^{+}$) is the energy of cation (anion) molecule that is calculated based on the optimized neutral molecule, and $E_{-}^{-}$ | $E_{+}^{+}$ | $E_{0}^{0}$ are the energy of the cation/anion/neutral that are calculated based on their optimized structure, respectively. Similarly, $E_{-}^{0}$ | $E_{+}^{0}$ is energy of the neutral molecule that is calculated based on the optimized cation (anion) molecule.

In most cases, absolute hardness serves as a prediction of stability properties, which can be predicted by the following formula (Cheung and Troisi, 2010):

(8) $$\begin{array}{l} \eta \;=\;\frac{1}{2}\left(\frac{\partial \mu }{\partial N}\right)=\frac{1}{2}\left(\frac{\partial^{2}E}{\partial N^{2}}\right)=\frac{IP-EA}{2} \end{array}$$

It is widely known that lower ionization potentials and higher electron affinities contribute to better efficiency of electron injection and exciton separation (Liu et al., 2010; Gui et al., 2012). Compared to NFA 1, other NFAs possess lower values of ionization potentials and NFA 8 possesses higher values of electron affinity, revealing that NFA 8 may do well in the transport ability of holes and electrons. Synthesizing the consequence of electron and hole, it is manifest that other NFAs possess lower reorganization energies than NFA 1, which means less barriers in the charge transfer and higher rates of electron and hole transfer. From Table 6, it is common for the designed acceptors to show slightly smaller values of stability than that of NFA 1. Taking steric hindrances into consideration, we suppose that there is little difference in stability properties between NFA1 and designed acceptors. Overall, it suggests that the introduction of different terminal units is favorable for the enhancement of hole–electron binding and molecular ionization, and would not cause significant variation in the stability of the acceptors.

## Conclusion

In this article, we design a class of novel non-fullerene acceptors based on dithienopicenocarbazole as the kernel module theoretically, and investigate the relevant qualitative parameters associated with J_SC_ and V_OC_ of these proposed molecules systematically. NFA 4 may be a good candidate due to its outstanding V_OC_ and obviously increased LUMO energy levels, as well as its slightly intense light-absorption ability. Though NFA 2, 7, and 8 do not highlight their value of V_OC_ like NFA 4, they exhibit superior optoelectronic properties of strong charge transfer and small energy loss, which corresponds to higher J_SC_, and could still be optimal acceptors with preferable photovoltaic performance. Therefore, we draw a conclusion that proper molecular modifications with the extension of electron-withdrawing units at the terminal group could make a conducive influence for OPV properties together with increased open circuit voltages, improved sunlight-absorbing ability, enhanced charge transfer, and decreased energy loss. It should also be noted that theoretical calculation is of enormous importance to molecule design to avoid unnecessary attempts. Finally, we believe this work could provide helpful guidelines for better and faster designing of DTPC-based photovoltaic molecules, and could shed light on the study of high-performance non-fullerene acceptors materials.

## Data Availability Statement

All datasets generated for this study are included in the article/Supplementary Material.

## Author Contributions

YL and HW conceived the idea and initiated this project. HW and JF wrote the manuscript. YL, NR, and HW contributed to fruitful discussions and supervision of the project. All authors discussed the results and commented on the manuscript.

## Conflict of Interest

The authors declare that the research was conducted in the absence of any commercial or financial relationships that could be construed as a potential conflict of interest.
