# Supplementary material for: Theoretical Design of Dithienopicenocarbazole-Based Molecules by Molecular Engineering of Terminal Units Toward Promising Non-fullerene Acceptors
Source: Front Chem. 2020 Nov 5;8:580252. doi: 10.3389/fchem.2020.580252 (PMC7674677; doi:10.3389/fchem.2020.580252)
Supplement: Supplementary file 1 [file Data_Sheet_1.DOCX]

Supplementary Material

# Supplementary Figures and Tables

**Figure S1.** The calculated optimum energy value of (PBT7-Th)_n_ (n= 1, 2, 3).

**Figure S2.** Optimized structure of PBT7-Th (simplified into (PBT7-Th)_3_).

**Figure S3.** Optimized structure of NFA 2,3,5-8 from the side view (a) and top view (b).

**Figure S4.** Hole and electron distribution for the S0🡒S1 transitions of NFA 2,7,8 (a), and heat maps associated with S0🡒S1 transitions for hole−electron overlap in various fragment of NFA 2,7,8 (b).

**Figure S5.** Charge density differences between ground state and first excited state and charge transfer distance D_CT_ (red arrows) for NFA 2,7,8 (a) (b) and half of NFA 2,7,8 (c) (d).

**Table S1.** Calculated HOMO energy levels (eV) of NFA1 with the functionals of B3LYP, PBE0 and MPW1B95, and corresponding experimental values.

**Table S2.** Calculated dihedral angles (in degrees) and bond lengths (in Å) for investigated NFAs.

**Table S3.** The HOMO orbital distributions (%) for investigated NFAs.

**Table S4.** The LUMO orbital distributions (%) for investigated NFAs.

**Table S5.** Calculated centroid distance D (Å), overlapping extent S_r_ Index, separation degree t of hole and electron, and coulombic energy E_coul_ (eV) for investigated NFAs.

## Supplementary Figures





**Supplementary Figure 1.** The calculated optimum energy value of (PBT7-Th)_n_ (n= 1, 2, 3).


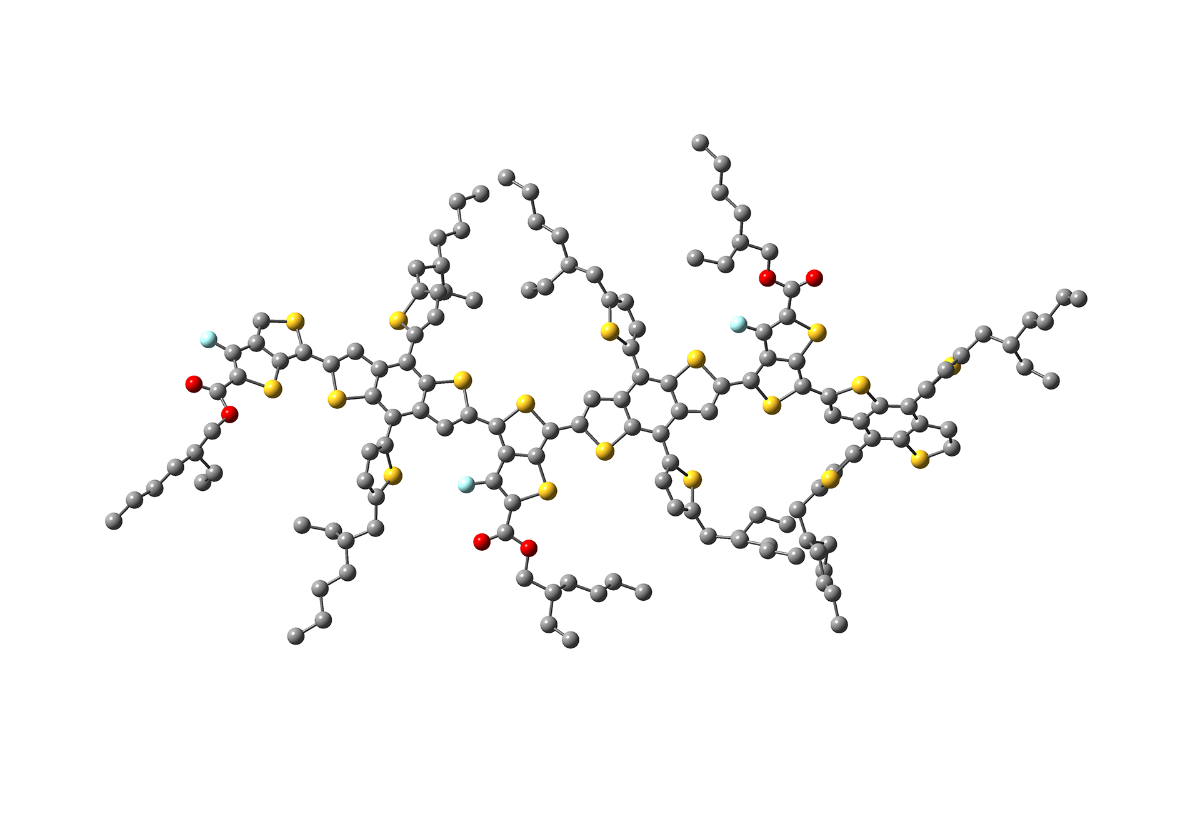


**Supplementary Figure 2.** Optimized structure of PBT7-Th (simplified into (PBT7-Th)_3_) (saturated H atoms are not shown).


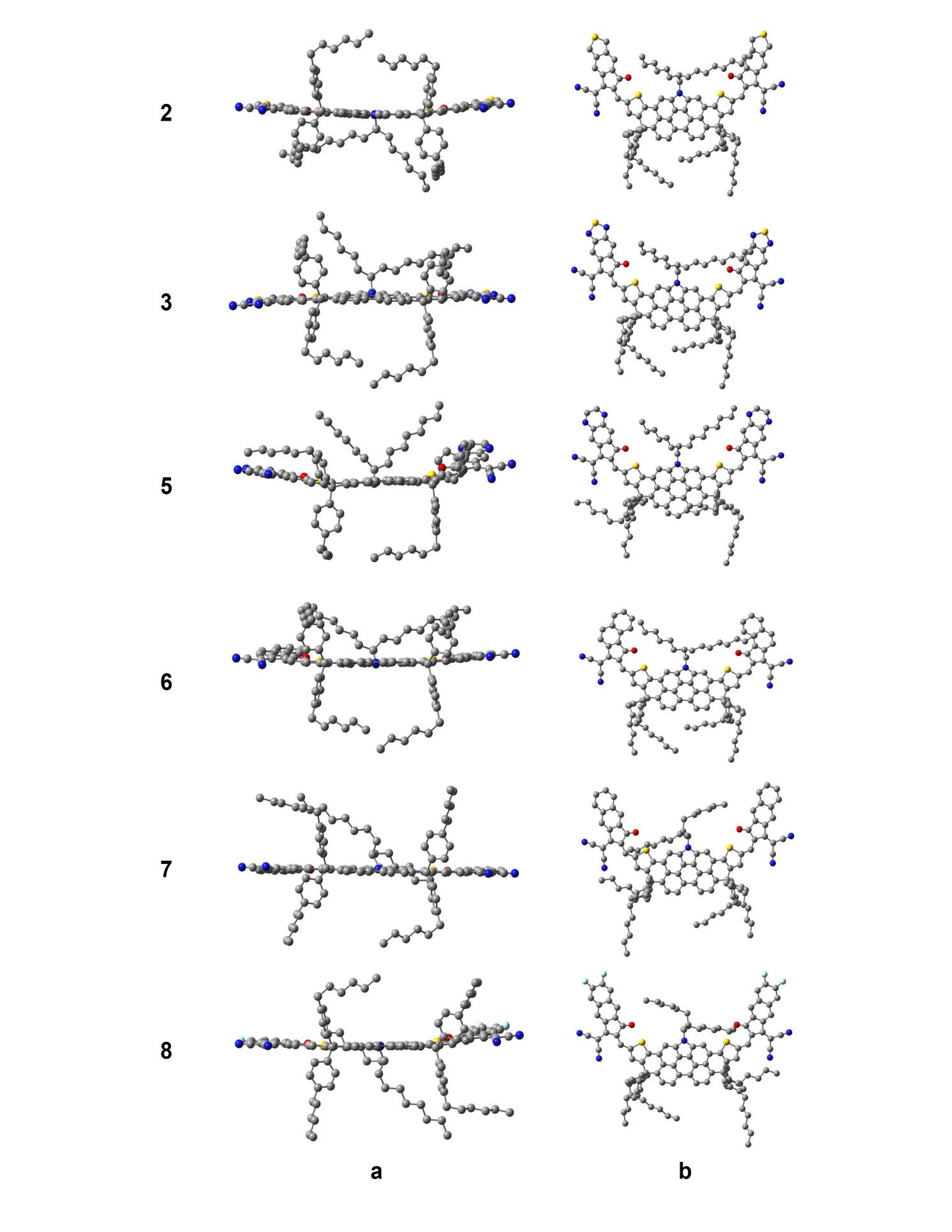


**Supplementary Figure 3.** Optimized structure of NFA 2,3,5-8 from the side view (a) and top view (b) (saturated H atoms are not shown).


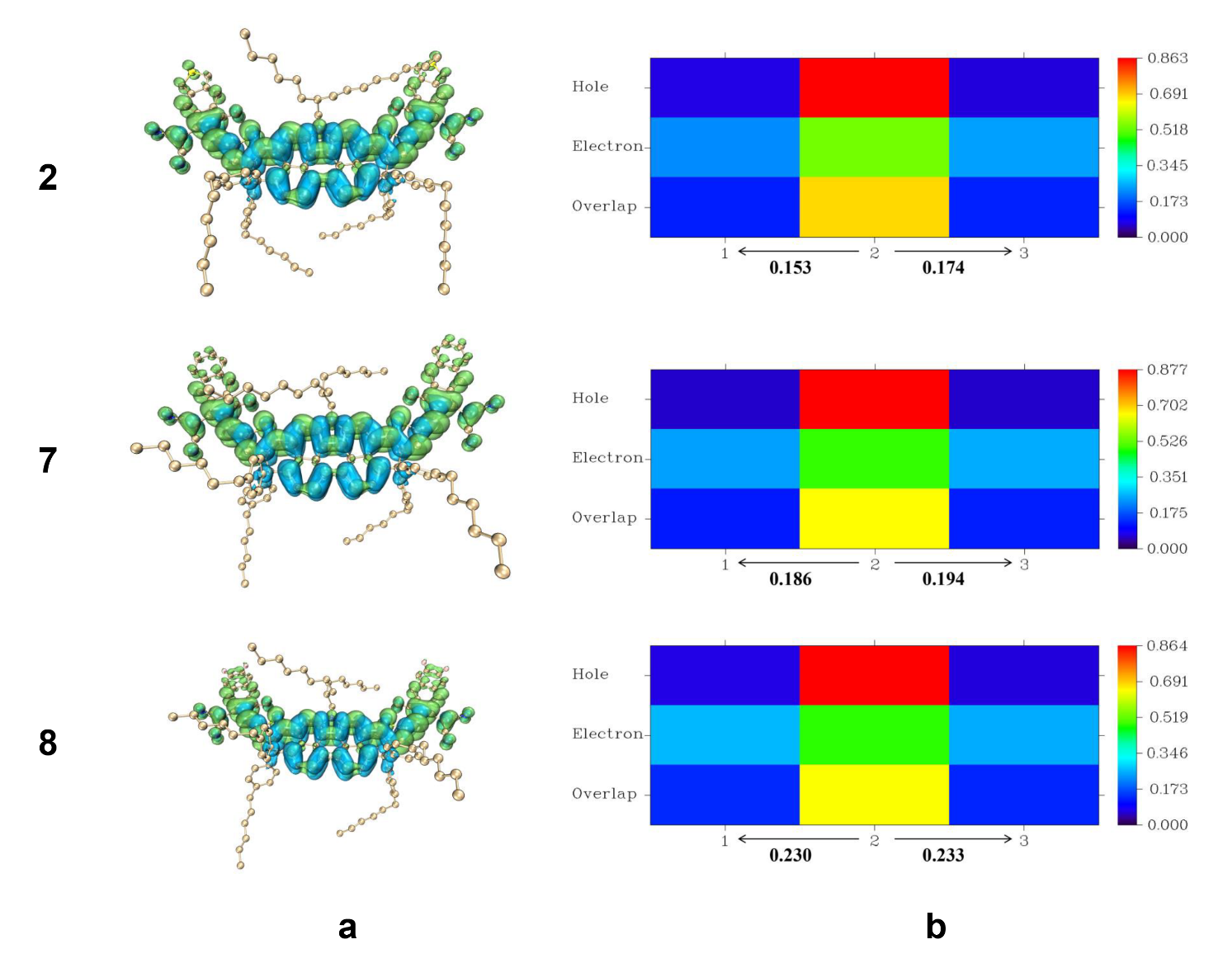


**Supplementary Figure 4.** Hole and electron distribution of NFA 2,7,8 (a) for the S0🡒S1 transitions, and heat maps associated with S0🡒S1 transitions for hole−electron overlap in various fragment of NFA 2,7,8 (b). Net electron transfer between fragments are also shown at the bottom of the figure.


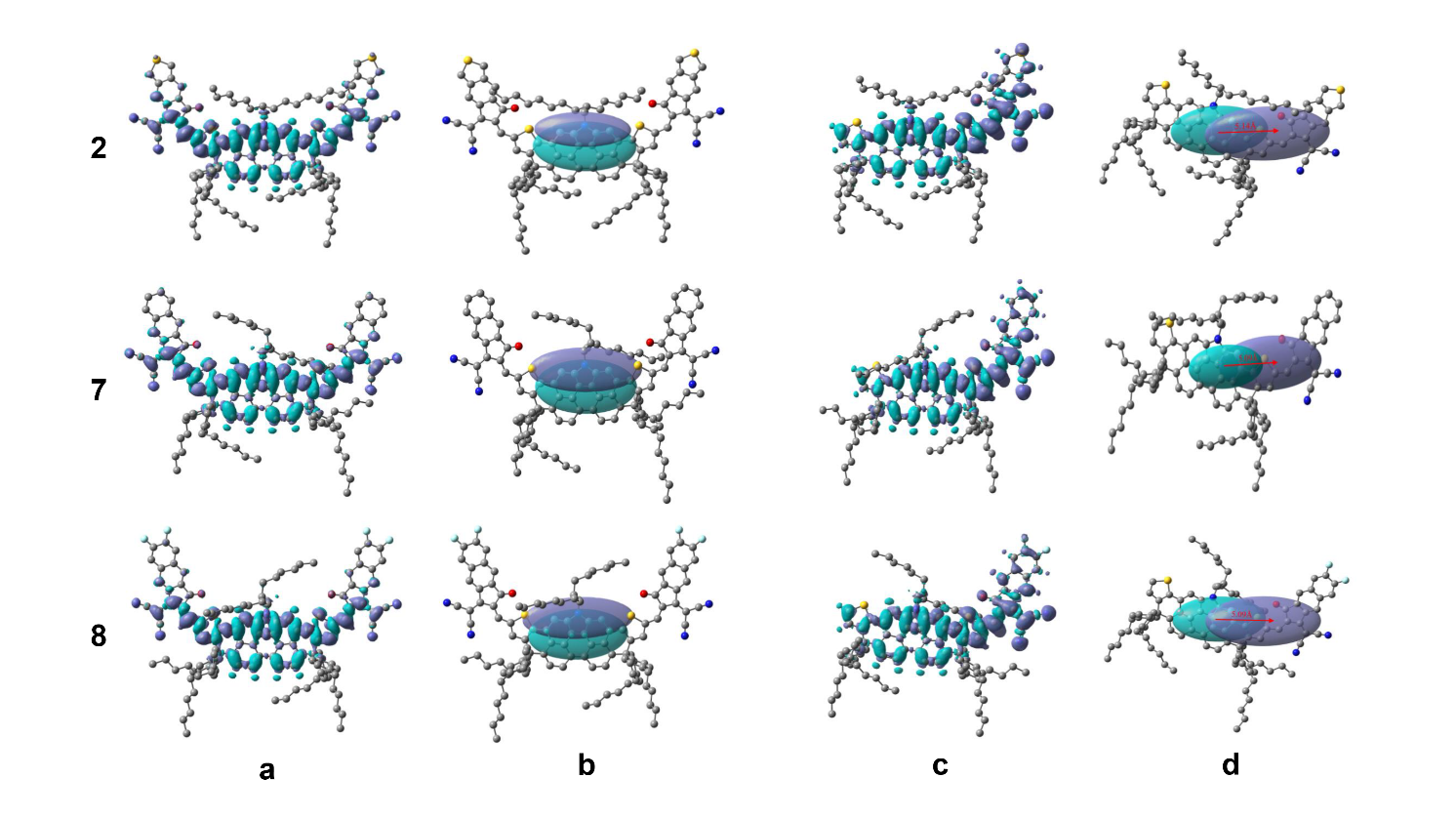


**Supplementary Figure 5.** Charge density differences between ground state and first excited state and charge transfer distance D_CT_ (red arrows) for NFA 2,7,8 (a) (b) and half of NFA 2,7,8 (c) (d).

## Supplementary Tables

**Supplementary Table 1.** Calculated HOMO energy levels (eV) of NFA1 with the functionals of B3LYP, PBE0 and MPW1B95, and corresponding experimental values.

| NFA1 | B3LYP  (20%HF_exc_) | PBE0  (25%HF_exc_) | MPW1B95  (31%HF_exc_) | EXP |
| --- | --- | --- | --- | --- |
| HOMO | -5.14 | -5.36 | -5.39 | -5.31 |

**Supplementary Table 2.** Calculated dihedral angles (in degrees) and bond lengths (in Å) for investigated NFAs.

| NFAs | α | Β | γ | φ | L_1_ | L_2_ |
| --- | --- | --- | --- | --- | --- | --- |
| 1 | 1.90 | 2.39 | 1.05 | 1.74 | 1.376 | 1.414 |
| 2 | 0.68 | 0.12 | 1.86 | 0.45 | 1.377 | 1.414 |
| 3 | 0.65 | 0.07 | 1.94 | 0.70 | 1.380 | 1.412 |
| 4 | 0.67 | 0.23 | 4.04 | 2.73 | 1.375 | 1.416 |
| 5 | 1.98 | 2.39 | 2.11 | 2.04 | 1.379 | 1.412 |
| 6 | 1.74 | 2.85 | 2.02 | 0.93 | 1.374 | 1.417 |
| 7 | 0.31 | 0.27 | 0.49 | 1.05 | 1.377 | 1.415 |
| 8 | 1.22 | 0.18 | 0.23 | 0.63 | 1.378 | 1.414 |

**Supplementary Table 3.** The HOMO orbital distributions (%) for investigated NFAs.

| NFAs | 1 | 2 | 3 | 4 | 5 | 6 | 7 | 8 |
| --- | --- | --- | --- | --- | --- | --- | --- | --- |
| DTPC-core | 84.77 | 83.31 | 82.62 | 84.68 | 83.32. | 85.34 | 84.01 | 83.87 |
| Acceptor | 7.75 | 8.44 | 8.76 | 7.64 | 8.48 | 7.34 | 8.02 | 8.06 |
| Acceptor | 7.49 | 8.25 | 8.62 | 7.68 | 8.20 | 7.32 | 7.96 | 8.07 |

**Supplementary Table 4.** The LUMO orbital distributions (%) for investigated NFAs.

| NFAs | 1 | 2 | 3 | 4 | 5 | 6 | 7 | 8 |
| --- | --- | --- | --- | --- | --- | --- | --- | --- |
| DTPC-core | 42.54 | 38.24 | 32.91 | 41.26 | 37.71 | 39.76 | 40.62 | 40.12 |
| Acceptor | 29.91 | 27.56 | 30.17 | 28.76 | 32.06 | 28.59 | 29.49 | 30.57 |
| Acceptor | 27.55 | 34.20 | 36.92 | 29.97 | 30.23 | 31.65 | 29.90 | 29.31 |

**Supplementary Table 5.** Calculated centroid distance D (Å), overlapping extent S_r_ Index, separation degree t of hole and electron, and coulombic energy E_coul_ (eV) for investigated NFAs.

| NFAs | 1 | 2 | 4 | 7 | 8 |
| --- | --- | --- | --- | --- | --- |
| D | 1.609 | 1.634 | 1.621 | 1.808 | 1.801 |
| S_r_ | 0.65 | 0.68 | 0.66 | 0.63 | 0.64 |
| t | -0.437 | -1.151 | -0.516 | -0.498 | -0.534 |
| E_coul_ | 2.36 | 2.38 | 2.35 | 2.25 | 2.30 |
